# Understanding the veterinary antibiotic flow in Malawi: complexities, gaps and needs

**DOI:** 10.3389/fvets.2024.1474307

**Published:** 2024-11-20

**Authors:** Amos Lucky Mhone, Dishon M. Muloi, Arshnee Moodley

**Affiliations:** ^1^Animal and Human Health Program, International Livestock Research Institute, Nairobi, Kenya; ^2^Department of Veterinary and Animal Sciences, Faculty of Health and Medical Sciences, University of Copenhagen, Copenhagen, Denmark; ^3^Institute of Infection, Veterinary and Ecological Sciences, University of Liverpool, Liverpool, United Kingdom

**Keywords:** antimicrobials, access, governance, practices, policy

## Abstract

**Introduction:**

Veterinary antibiotics are essential for maintaining animal health and welfare, however, small-scale farmers in Malawi face challenges in accessing them due to limited availability, affordability, and long distances to rural drug retailers.

**Methods:**

This study mapped the veterinary antibiotic distribution chain, examined the governance structure of the chain, and analyzed access and usage practices among stakeholders in Malawi. Data were collected through focus group discussions (*n* = 15), key informant interviews (*n* = 6) and individual interviews (*n* = 189).

**Results:**

The key stakeholders identified included regulators, local pharmaceutical manufacturers, wholesalers, veterinary clinics, veterinary retail shops, animal health practitioners, and farmers. The distribution of veterinary antibiotics was characterized by both formal and informal pathways for importing and distributing veterinary medicines. Additionally, there were issues with antibiotic mishandling such as improper storage on open shelves in direct sunlight and disposal in pit latrines. There was a marked lack of proper antibiotic dispensation training among veterinary medicine shop attendants, and in terms of regulation, there were gaps in coordination and overlapping mandates among regulatory authorities hindering effective regulation.

**Discussion:**

Regulatory agencies need to strengthen oversight of veterinary antibiotics, conduct trainings on antibiotic stewardship with various stakeholders, and enhance public-private partnerships to better manage the informal pathways for importing and distributing veterinary medicines. This multi-sectoral approach aims to ensure responsible use and improve the pharmacovigilance of veterinary antibiotics.

## 1 Introduction

The livestock sector contributes 22.4% to the agricultural gross domestic product in Malawi (1). Despite its significant contribution, this sector receives minimal investment. For example, over the last decade, only 0.8% of the annual government budget has been allocated to this sector, resulting in poor animal health and veterinary service delivery (1). Furthermore, the sector is plagued by diseases crucial to production and the economy, such as African swine fever, Foot and Mouth Disease, East Coast Fever and Newcastle Disease, and common zoonotic infections including rabies, bovine tuberculosis, brucellosis, cysticercosis, and human African trypanosomiasis (2–4). Veterinary antibiotics are key in combatting some of these animal-specific and zoonotic infections, significantly benefitting animal health, welfare and food production. With Malawi’s urban population rising, the demand for animal-source foods has increased, resulting in intensified livestock production and consequently a growing demand for veterinary antibiotics (1).

Malawi’s pharmaceutical market is rapidly expanding and is expected to grow by 3.2% in 2024 reaching a value of US$116 M by 2028 (5). Effective management of the veterinary antibiotic supply chain is crucial for ensuring the availability, affordability and accessibility of essential medicines including veterinary antibiotics (6). This management is a key component of antimicrobial stewardship (AMS), as it ensures that medicinal products are available when needed, at the right price, quality, and quantity (7). This aligns with Malawi’s Antimicrobial Resistance Strategy (2017–2022), which emphasizes strengthening the management of the veterinary antibiotic supply chain to guarantee continuous access to high-quality antimicrobials (8). Mapping this distribution is vital for identifying access challenges, potential misuse or overuse, monitoring antibiotic quality, and enhancing transparency and coordination among stakeholders. Furthermore, it is crucial to implement policies for responsible antibiotic use, ensure access to essential medicines, and improve veterinary services. This includes better regulatory frameworks and establishing antibiotic use/consumption surveillance (9–11).

In contrast to the streamlined medicine distribution in Malawi’s public hospitals, managed by a single agency, the Malawi Central Medical Stores Trust (6), the flow of veterinary antibiotics involve a complex network of public and private sector stakeholders. Few studies have been conducted to understand the intricacies of veterinary antibiotic distribution in the country, including the roles and responsibilities of different stakeholders and the challenges they face. Kainga et al. (12) highlighted the poor knowledge, negative attitudes, and inadequate practices among retail veterinary drug dispensers regarding antimicrobial use and antimicrobial resistance in Malawi’s major cities. Khuluza et al. (13) reported only a 71.1% availability of essential veterinary antibiotics at the wholesale pharmacy level. Similarly, Mankhomwa et al. (14) observed that the veterinary shop environments were highly permissive, with customers never being refused sales, even without a prescription. If a farmer was unfamiliar with the medication they needed, the shop attendant would dispense based on their knowledge of the medicines in stock. This study aimed to (1) map the routes through which veterinary antibiotics pass from production or importation to the end users, e.g., farmers, (2) identify key stakeholders and their roles within the veterinary antibiotic distribution, (3) explore existing governance structures, and (4) investigate the challenges faced by various stakeholders.

## 2 Materials and methods

### 2.1 Study area, design, and ethical approval

**Figure 1:**
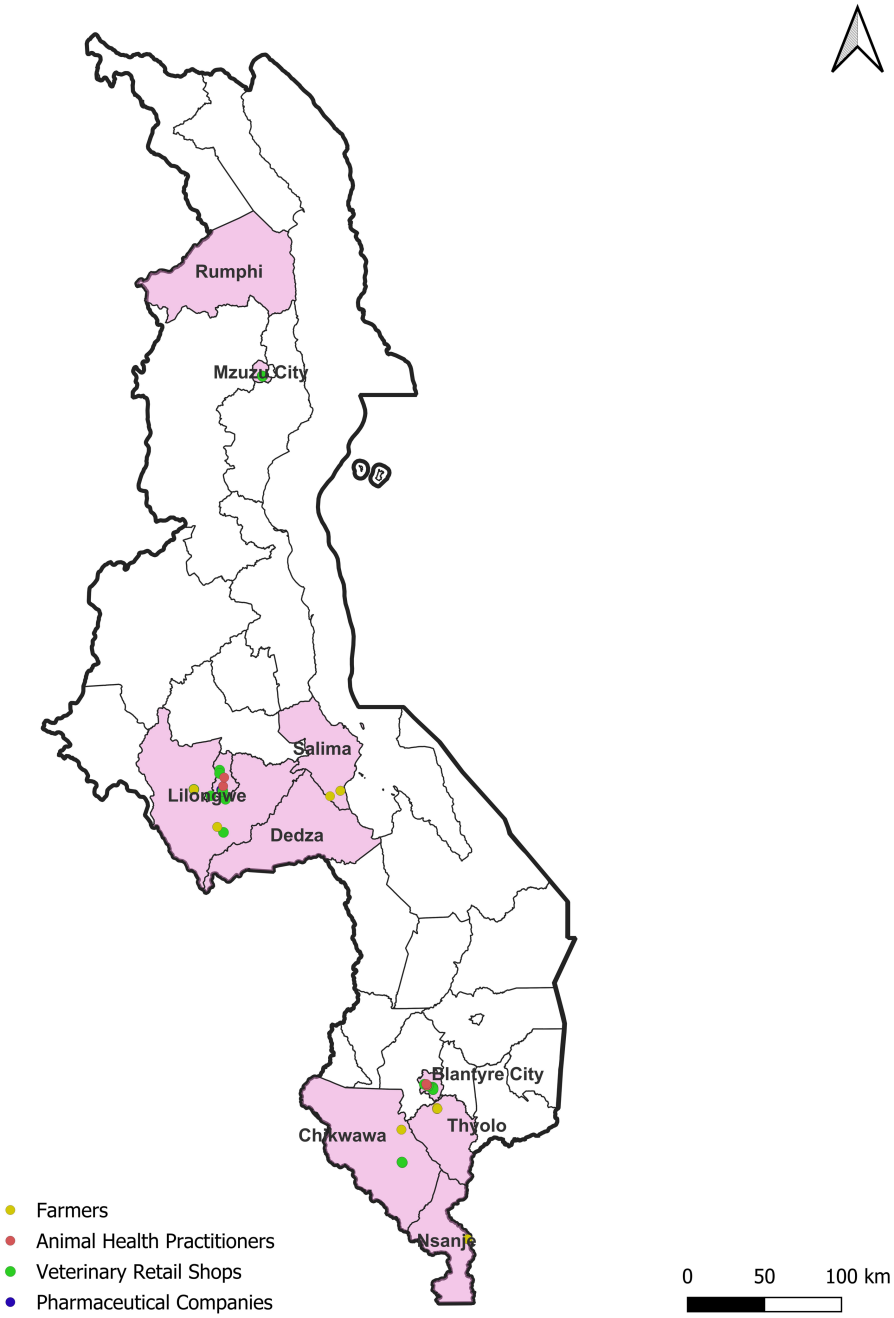
Geographic distribution of farmers, animal health practitioners, veterinary retail shops and pharmaceutical companies participating in the study.

A cross-sectional study was carried out targeting stakeholders along the veterinary antibiotic flow in Malawi with a focus on three main cities: Mzuzu, Lilongwe and Blantyre (Figure 1). These cities are the administrative and commercial centers for the northern, central, and southern regions of Malawi, respectively. They account for 90% of the Malawian pharmaceutical industry including wholesale distributors, veterinary retail shops, and veterinary clinics (12). While mixed farming dominates across all three regions (15), some variations exist. For instance, dairy and beef cattle farming is common in the peri-urban areas surrounding Blantyre, located within the Shire Highlands in the southern region (2). The central region, in contrast, boasts extensive and intensive poultry and pig farming (16). Finally, both the central and northern regions are predominated by aquaculture and small ruminant farming (12, 17). Malawi does not have farm registry system, therefore we mapped individual farmers, in Lilongwe (poultry and pigs), Blantyre (Dairy and Beef), and Mzuzu (goats and fish) with the help of local veterinary officers in the 3 major cities and the surrounding districts. Afterwards farmers were randomly selected to be included in the study, but we sought to purposively ensuring we covered the whole geographical area of the district and captured data on both subsistence farmers and those engaged in small-scale intensive farming. Snowball techniques was applied to recruit veterinary retail shops and animal health practitioners. This study was carried out between May and June 2023. Ethical approval was obtained from the International Livestock Research Institute (ILRI) Institutional Research Ethics Committee (ILRI-IREC) (project reference: ILRI-IREC2022-33) and research permits obtained from Malawi Government’s Department of Animal Health and Livestock Development (DAHLD) (project reference: DAHLD/AHC/01/2023/07).

### 2.2 Data collection

**Figure 2:**
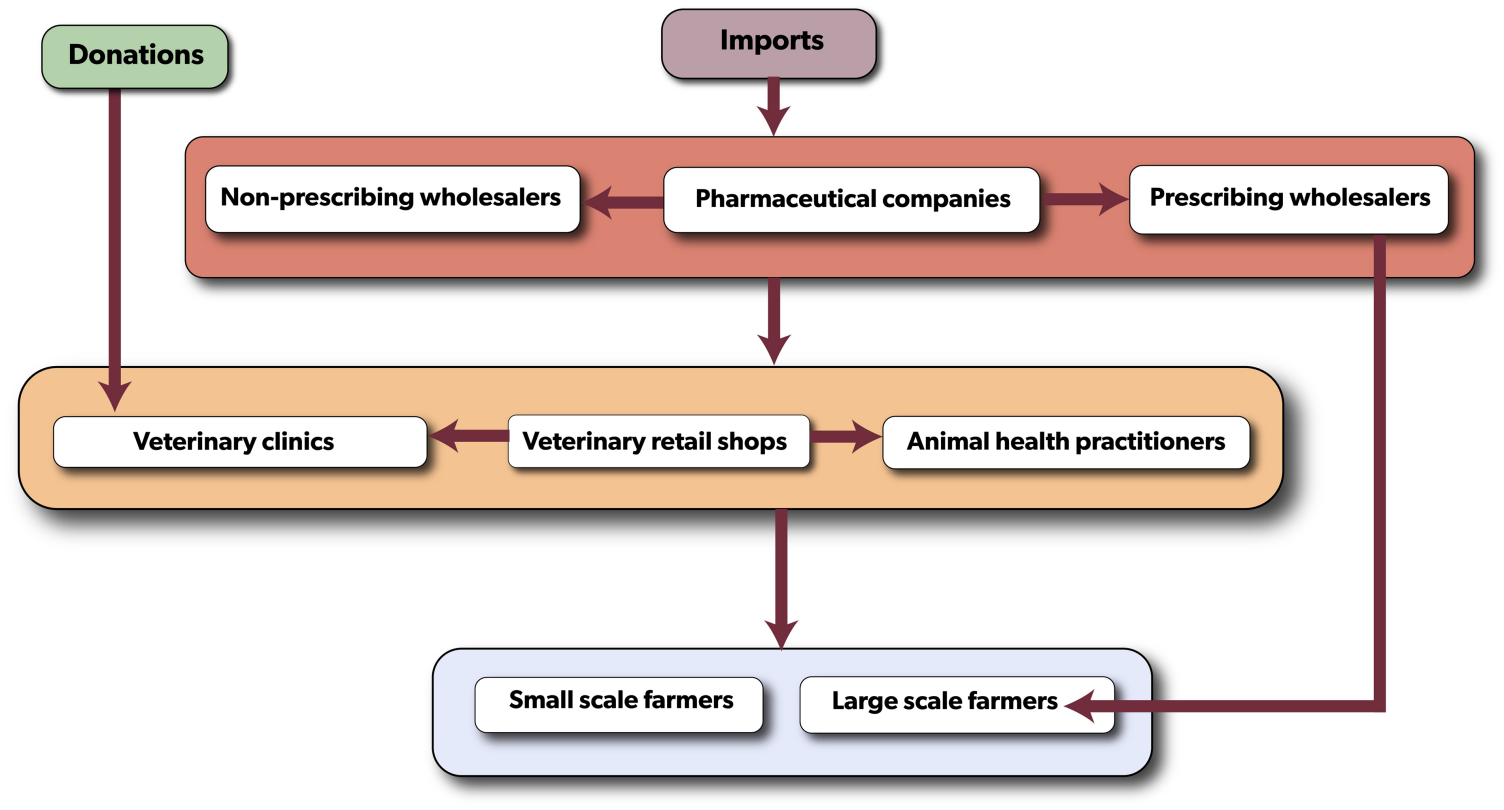
The conceptual map of the veterinary antibiotic flow in Malawi illustrating the various levels of the distribution. Colored boxes denote the different levels of the flow and the stakeholders involved at each level.

A desk review using published materials and initial interviews with regulatory authorities was conducted to develop a conceptual map for the flow of veterinary antibiotics in the country (Figure 2). This framework provided a basic understanding of distribution flow, identified key stakeholders, and served as a guide for further discussions and interviews. Three distinct methods were used to obtain primary data: individual interviews, focus group discussions (FGDs), and key informant interviews (KIIs) (Table 1). Semi-structured questionnaire for individual interviews, and FGDs guide were designed, tested, and validated in Kenya, and were previously used in a similar study in Nairobi, Machakos and Kajiado Counties of Kenya (18). They were designed in English and were verbally translated into the local Chichewa language during administration. To ensure proper translation of the questions, local interviewers/facilitators who were fluent in Chichewa and with background of veterinary medicine and public health were used. FGDs were organized with farmers, animal health practitioners, and veterinary retail shop owners/staff to collect both qualitative and quantitative data. A total of 17 FGDs were carried out with farmers (11), veterinary retail shops (3) and animal health practitioners (3), with an average of 10 participants per group. Participants were asked to explain their operational processes, map the antibiotic flow based on their knowledge, and outline stakeholder responsibilities and obligations. Regulatory themes such as licensing, pricing, regulations, and legislation were also investigated. In addition, individual interviews were conducted with FGD participants using a standardized semi-structured questionnaire to gain further insights into their views on the governance frameworks and the distribution of veterinary antibiotics. To supplement the information collected from FGDs, KIIs were conducted with representatives from the Malawi Revenue Authority, pharmaceutical companies, wholesalers, the Pharmaceutical Society of Malawi, the Pharmacy Medicines Regulatory Authority (PMRA), and the Department of Animal Health and Livestock Development (DAHLD). The KIIs followed a similar approach to the FGDs but included additional questions about participants’ roles and potential impacts on antibiotic flow, pharmaceutical products registration, antibiotic quality control, market authorization, product recall, product licensing and import permits. Notebooks, flip charts, audio recordings, Open Data Kits (ODK), focus groups, and key informant interviews were utilized to document the qualitative data collected.

**Table 1:** Overview of stakeholders interviewed in the study.

Type of interview	Category	Number of participants
FGDs and individual interviews	Farmers	107
	Veterinary retail shop workers/owners	35
	Animal Health Practitioners	38
Key informant interviews	Regulatory authorities	3
	Pharmaceutical companies	1
	Wholesalers	3
	Pharmaceutical Society of Malawi (PHASOM)	1
	Malawi Revenue Authority	1

### 2.3 Data management and analysis

Audio recordings were reviewed, and relevant information was transcribed and transferred to NVIVO version 14.23.0 for thematic qualitative analysis. Key themes related to the study objectives were extracted, including veterinary antibiotic flow, stakeholders’ challenges, antimicrobial use, antimicrobial policy challenges, recommendations, and practices in antimicrobial access and use. Data from Excel sheets in ODK were cleaned and transferred to GraphPad Prism version 10.0.1 for descriptive analysis, focusing on the knowledge, attitudes, and practices regarding the veterinary antibiotic flow among animal health practitioners, veterinary retail shops, and livestock farmers (10, 19).

## 3 Results

### 3.1 Structure and flow of veterinary antibiotics in Malawi

**Figure 3:**
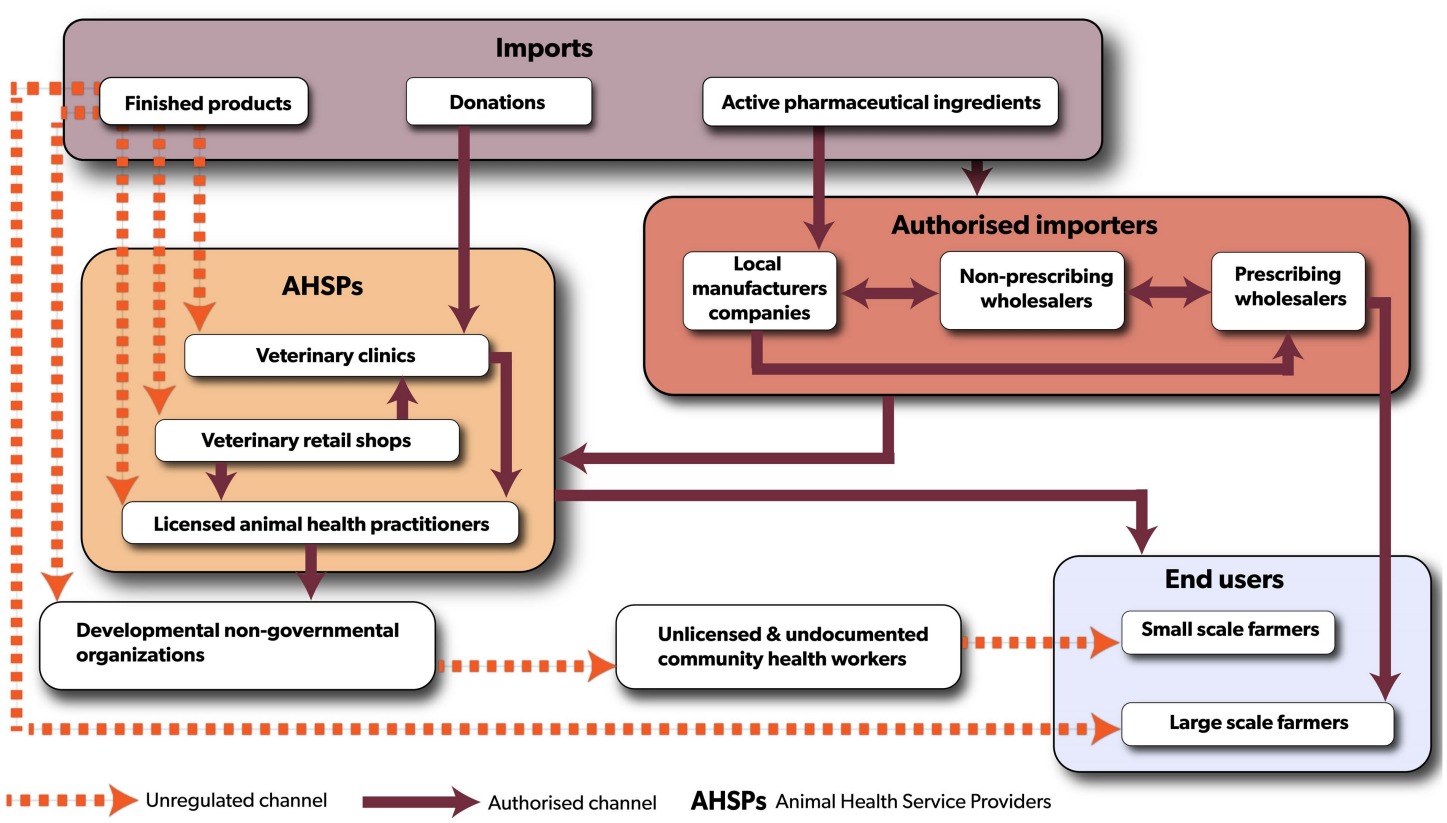
The flow of veterinary antibiotics in Malawi is depicted with solid lines representing formal regulated distribution channels, and red dotted lines indicating unregulated routes.

The veterinary antibiotic distribution chain in Malawi involves both public and private sector actors (Figure 3). In the public sector, key stakeholders include the Pharmacy Medicines Regulatory Authority (PMRA), the Department of Animal Health, and Livestock Development (DAHLD), the Department of the Registrar General, and city/district councils. These bodies regulate various aspects of the distribution of veterinary antibiotics in Malawi. The private sector predominantly comprises of pharmaceutical companies operating outside Malawi, as the country relies entirely on importing veterinary antibiotics, either as finished pharmaceutical products (85%) or as active pharmaceutical products (15%). Only registered importers with valid pharmaceutical wholesale licenses are permitted to import antibiotics. These include (1) prescribing wholesalers who distribute veterinary antibiotics in bulk and prescribe to large-scale farms, (2) non-prescribing wholesalers who only sell veterinary antibiotics in bulk, and (3) local pharmaceutical companies that manufacture veterinary antibiotics using imported active pharmaceutical products and/or repackage finished pharmaceutical products for retail. Veterinary clinics and veterinary retail shops, source antibiotics through importers and cater for varying customer needs such as selling veterinary antibiotics in smaller quantities. Animal health practitioners who provide extension and animal health services to livestock farmers, purchase veterinary antibiotics from wholesalers and retailers for use on farms. Farmers typically buy antibiotics from veterinary retail shops or animal health practitioners. During animal disease outbreaks of bacterial nature, large-scale farmers needing substantial quantities of veterinary antibiotics may procure them directly from prescribing wholesalers, subject to PMRA’s approval.

### 3.2 Governance of the veterinary antibiotic flow in Malawi

#### 3.2.1 Regulatory framework

The PMRA is tasked with overseeing the importation, manufacture, distribution, sale, and exportation of veterinary antibiotics as mandated by the PMRA Act of 2019 (20). According to the Veterinary and Para Veterinary Act of 2001 (21), the DAHLD is responsible for regulating veterinary surgeons and veterinary paraprofessionals, and licensing of veterinary clinics. The Department of the Registrar General manages business registrations for veterinary antibiotics business entities under the Business Registration Act of 2013 (22), while city/district councils issue business permits as per the Businesses Licensing Act of 2013 (23).

#### 3.2.2 Product registration, importation and exportation

In Malawi, only authorized importers with active permits are allowed to import antibiotics, and they must comply with national, regional and international registrations such as the Southern African Development Community Medicines Regulatory Harmonization (ZAZIBONA initiative), United States Food and Drug Administration, or European Medicines Agency. The ZAZIBONA initiative was established to tackle shared issues such as application backlogs, long registration times, high staff turnover, limited financial resources, and insufficient capacity for assessing products like biologicals and biosimilars. The initiative concentrates on dossier assessments and good manufacturing practice inspections (24). The United States Food and Drug Administration regulates the drug approval process, requiring medicines to meet strict standards before they are marketed. The agency also monitors post-market safety, oversees manufacturing practices, and fosters innovation through scientific research. Additionally, the FDA collaborates internationally to harmonize standards and improve global public health (25). The European Medicines Agency (EMA) is an EU agency responsible for the scientific evaluation, supervision, and safety monitoring of medicines in Europe. It collaborates with national regulatory bodies to ensure that medicines meet high standards of quality, safety, and efficacy. The EMA oversees the centralized drug approval process, allowing companies to market their products across the EU with one application, and plays a key role in pharmacovigilance (26). These importers must meet import requirements including possession of a certificate of the pharmaceutical product, a valid manufacturing practice certificate, and a certificate of analysis from the manufacturer. Moreover, they must have an authorized local agent and distributor or market authorization holder for the registered pharmaceutical products. Lastly, each authorized importer must annually provide an updated list of their manufacturers to PMRA, for inspections (27).

#### 3.2.3 Veterinary antibiotics business

To operate a veterinary retail store in Malawi, the owner must receive approval from PMRA. The applicant must submit an application registration form, a sketch of the premises layout, a copy of their taxpayer identification number issued by the Malawi Revenue Authority (MRA), copies of relevant veterinary professional qualifications, a signed contract or commitment letter for employed persons, a copy of business registration from Registrar of Businesses, and a copy of the application fee receipt from PMRA. In veterinary retail shops or clinics, only an authorized prescriber is permitted to dispense veterinary antibiotics to farmers. According to the PMRA Act of 2019 (20)and the Veterinary and Para-Veterinary Act of 2001 (21), an “authorized prescriber” is defined as a veterinary surgeon or a para-veterinary surgeon who holds a degree, diploma, certificate or other qualification recognized by the Board of Veterinary Surgeons. The individual must satisfy the Board of Veterinary Surgeons that they have sufficient knowledge of veterinary science, an adequate knowledge of the English language, and are of good character, making them fit and proper to be registered.

#### 3.2.4 Antibiotic quality control

The National Medicine Quality Control Laboratory, part of the PMRA, is responsible for regulating the quality of veterinary antibiotics before market authorization and conducting continuous post-market surveillance. According to the PMRA, the laboratory routinely performs several tests, including identification, friability, disintegration, and uniformity tests, using techniques such as High-performance Liquid Chromatography and spectrophotometry. The cost of routine drug analysis per batch ranges from $36–$300. Licensed importers bear the initial testing costs when applying for market authorizations, while routine post-market testing is covered by PMRA as part of ongoing post-market surveillance (28).

### 3.3 Challenges with the veterinary antibiotic distribution, governance and policy gaps in Malawi

Our interviews with stakeholders revealed that veterinary retail shop owners, veterinary clinics, animal health practitioners, and farmers were directly importing veterinary antibiotics, in violation of the PMRA Guidance on importation and Exportation of Medicinal Products and Active Pharmaceutical Ingredients (27), that designated only wholesalers and local manufacturers as authorized importers. We identified gaps in coordination and overlapping mandates among antibiotic regulatory authorities. In a KII, it was reported that this lack of coordinated mechanisms for veterinary antibiotic registrations and inspections potentially leads to the surge of unregistered products, an increase in expired/inactive/outdated market licenses, delays in issuing importation permits, and unrecorded imported veterinary antibiotics in national import data, which negatively affects continuous post-market AMU surveillance. During FGDs, pharmaceutical business owners indicated that delays in issuing permits and conducting inspections impacted distribution timelines, availability, and quality of antibiotics. Additional challenges included the scarcity of foreign currency affecting imports and geographical barriers limiting access to essential antibiotics. The PMRA is currently reviewing guidelines for registration, importation, and inspections of veterinary medicines to strengthen regulations. However, the regulatory authority is facing limited funding and human resources for enforcement. Finally, Malawi lacks an essential veterinary medicines list, which would improve accessibility and affordability of veterinary antibiotics.

### 3.4 Access, use and practices around antibiotic use by antibiotic retailers and end users

#### 3.4.1 Access to veterinary antibiotics and antibiotic-related information

Veterinary retail business owners reported that demand for veterinary antibiotics dictates the stocking levels and pricing. According to the FGDs, Malawi heavily relies on imported veterinary antibiotics due to the country’s limited production capacity. This reliance hampers the steady supply of high-quality and sufficient quantities of veterinary antibiotics, exacerbated by both economic and regulatory issues. Due to a shortage of foreign currency and the high cost of veterinary antibiotics, importers only bring a few batches into the country. Additionally, delays in market authorizations, licensing of sales locations, import permits, and inspections by regulatory authorities prolong the time it takes for businesses to become operational, limiting access to quality antibiotics. Farmers reported being unable to afford certain veterinary medicines due to high prices, leading them to use alternative herbal remedies such as cinchona bark and *aloe vera*, which they also believe are effective. Access to antibiotic information was primarily obtained from regulatory bodies, according to veterinary retail stores (74.3%, *n* = 26) and AHPs (65.8%, *n* = 25) (Supplementary Table S1). However, they lacked knowledge on the correct disposal methods of expired or unused antibiotics and called for better information exchange channels. Most farmers had limited knowledge about antibiotics, particularly regarding their identification, usage, and disposal (76.6%, *n* = 82), as well as about the regulatory authorities overseeing them (96.3%, *n* = 103) (Supplementary Table S1). Farmers also stated that community networks with other farmers provided most of their information about antibiotics, their usage, and disposal. These networks consisted of either neighboring farmers who kept similar animals, or a farmers cooperative group that kept the same animals. They shared information on their livestock at one-on-one meetings, funerals, tradition authority meetings or during cultural festivals. If one farmer used a certain antibiotic brand and his/her production improved, others were likely to use the same antibiotic. As such, these networks influenced the types of antibiotics commonly purchased by farmers.

#### 3.4.2 Antibiotic use

Over two-thirds of veterinary retail stores reported prescribing and dispensing veterinary antibiotics for prophylaxis (68.6%, *n* = 24), primarily for poultry (40.2%, *n* = 27) and pigs (28.3%, *n* = 19) (Supplementary Table S1). Almost all veterinary retail stores (85.7%, *n* = 30) reported selling veterinary antibiotics based on clinical signs and symptoms provided by farmers. AHPs primarily treated animals based on clinical symptoms (73.7%, *n* = 28) rather than diagnostic tests, with over half (47.4%, *n* = 18) prescribing preventative therapy, mainly for poultry (31.6%, *n* = 12) and pig farms (18.4%, *n* = 6) (Supplementary Table S1). During the FGDs, participants expressed concerns about the turnaround time for test results. AHPs indicated that when they send samples to a veterinary lab for testing, the extended turnaround time often leads them to prescribe veterinary medications without laboratory confirmation.

#### 3.4.3 Storage, and disposal practices

Retailers (88.6%, *n* = 31), animal health practitioners (31.6%, *n* = 12), and farmers (86.9%, *n* = 93) primarily stored veterinary antibiotics on open shelves (Supplementary Table S1). Refrigerators were mainly used by veterinary retail shops for animal vaccines, such as Newcastle disease vaccines and diagnostic tests such as tuberculin, both of which require an uninterrupted cold chain. Veterinary retail shops disposed expired antibiotic packages by burning in rubbish pits (54.3%, *n* = 19), while animal health practitioners (86.8%, *n* = 33) and farmers (86.9%, *n* = 96) often discarded them in pit latrines (see Supplementary Table S1). According to the FGDs, the recommended procedure involves sending expired packages to regulatory authorities for disposal; however, this procedure is currently not functional, leading many to resort to using bins, burning, and latrines for disposal.

## 4 Discussion

The study identified unregulated channels through which antibiotics are distributed, ultimately reaching the end users. These channels include veterinary retail shops, animal health practitioners, and farmers directly importing veterinary antibiotics instead of using authorized importers. These unregulated pathways pose a challenge as the products entering the country are not monitored for quality and are not recorded in AMU surveillance reports. Poupaud et al. (10) reported similar unregulated antibiotic channels in Laos, where farmers were illegally importing veterinary antibiotics from Thailand, Vietnam, and China. Similarly, in Uganda, Dione et al. (9) found that animal health practitioners, and farmers were accessing veterinary antibiotics directly from wholesalers instead of retailers. There is a need to strengthen regulations and enforcement mechanisms to ensure veterinary antibiotics are only accessible to end users through licensed importers and distributors. This can be done by (1) implementing robust tracking and tracing systems to monitor the movement of antibiotics, ensuring their quality, and monitoring AMU, (2) conducting education campaigns to raise awareness about the risks of accessing veterinary antibiotics through unregulated channels and the importance of responsible antibiotic use, is essential, (3) imposing strict penalties, including fines or loss of business licenses, for those selling and using antibiotics with are imported through unregulated pathways, (4) and involving law enforcement agencies to identify and shut down illegal antibiotic distribution networks, especially in areas with high rates of unregulated sales.

The study identified gaps in the coordination and enforcement of regulations governing the flow of veterinary antibiotics in Malawi. Weak coordination undermines efforts to effectively regulate the availability and access to antibiotics (29). Uncoordinated post-market surveillance, supply chain monitoring, and response mechanisms may impede the early detection of poor-quality veterinary antibiotics before they reach end users, increasing the risk of antibiotic resistance development (30). Mankhomwa et al. (14) reported that overlapping mandates among antibiotic regulators have led to gaps in post-market surveillance, resulting in some restricted antibiotics being sold to farmers. For example, colistin, a drug considered critically important and banned from sale in veterinary retail shops in Malawi, is commonly sold to farmers through retail outlets (14). Similarly, Nayija et al. (31) noted that gaps in coordination among antibiotic regulators have led to veterinary medicines business licenses being issued to unqualified individuals, as well as limited or non-existent quality assessment and registration of veterinary antibiotics. There is a need to (1) clearly demarcate the responsibilities of different government institutions to avoid clashing mandates, (2) establish a formal inter-agency task force that includes representatives from all relevant bodies, including environmental, human health, and trade sectors. This task force should meet regularly to streamline communication and improve oversight of veterinary antibiotic distribution and use. (3) Adoption of digital platforms for sharing data on antibiotic use, resistance patterns, and regulatory practices can facilitate coordinated action on optimizing antibiotic use in the animal sector. (4) Seeking bilateral partnerships with agencies such as the SADC, and EU to provide expertise, training, and resources for governance, surveillance and regulation systems.

Animal health practitioners primarily relied on clinical symptoms rather than diagnostic tests, to make diagnosis and prescribe treatment due to extended turnaround time of tests at the veterinary laboratory. Previous studies have demonstrated that some animal health practitioners prescribe antibiotics without laboratory confirmation of the pathogen and its susceptibility to antibiotics (32, 33). This may be due to lack of human, technical and financial capacities at the national veterinary laboratories resulting into extended turnaround time of tests (34), as well as lack of the standard treatment guidelines, and high cost of sending samples to the laboratory (35). This practice could lead to misdiagnosis prolonging suffering for the animals, worsening of the condition or death, and the empirical treatment could accelerate the development of AMR. There is need to strengthen veterinary laboratory infrastructure, by increasing resources, staff, and technology can reduce the turnaround time for test results, develop standard treatment guidelines and create a national database that allow for real-time sharing of disease surveillance data can provide practitioners with information on common pathogens in their area, helping guide treatment decisions without immediate diagnostics, sensitize for country wide adoption of on-farm diagnostic tools can reduce reliance on clinical symptoms alone. For example, Point of Cow Mastitis diagnostic Kit (36), and porcine delta coronavirus rapid test kit (37). These tools provide rapid, on-site diagnostics, helping practitioners make evidence-based decisions.

AHSPs and farmers indicated that they store veterinary antibiotics on open shelves and dispose of expired antibiotics in pit latrines. This open-shelf storage exposes antibiotics to light, heat, and moisture, which can degrade the active ingredients, reducing their efficacy and increasing the risk of antibiotic resistance (38). A study in Blantyre found that between 19 and 44% of amoxicillin, ciprofloxacin, flucloxacillin and sulfadoxine/pyrimethamine in drug stores were substandard due to poor storage (39). When antibiotic residues are disposed of in the environment via pit latrines, they can be transported through surface runoff and infiltrating water into surface water bodies or groundwater. This can lead to the emergence, selection and persistence of antimicrobial resistance genes (ARGs) (18). Therefore, correct storage and disposal of antibiotics are essential. The government, in partnership with the pharmaceutical industry, could implement a reverse logistics program to facilitate the safe disposal of unused or expired veterinary antibiotics, allowing stakeholders to return medications at designated collection points, such as veterinary retail shops or wholesalers, for correct disposal through incineration or other environmentally friendly methods.

Most antibiotic shop attendants in our study were not trained in animal health, which contravenes the PMRA guidelines for operating a veterinary retail shop, which states that a veterinary retail shop store should be attended to by a full-time veterinary professional (40). Similar observations were made by Kainga et al. (12) among veterinary drug dispensers in Malawi. In Tanzania, 72.5% of veterinary paraprofessionals in veterinary shops admitted to not having undergone formal training on antibiotic dispensing, antimicrobial use and resistance (41). These untrained, unlicensed attendants dispense veterinary antibiotics without a prescription or need, such as for viral infections, and may provide incorrect advice on dosages and/or duration of antibiotic therapy. This misuse can lead to treatment failure, adverse reactions, or the development of antibiotic resistance. Therefore, (1) enforcing existing regulations on the sale of veterinary antibiotics or introducing new regulations that ensure veterinary antibiotics are sold only by licensed prescribers is necessary. (2) Implementing mandatory training programs for drug shop attendants on appropriate antibiotic use and farmer case handling, and strengthening monitoring of veterinary antibiotics sales, can help identify areas for targeted interventions. (3) There is also need for recognize and reward shops that follow best practices in antibiotic sales, promoting them as model establishments.

Lastly, all stakeholders indicated that there was little to no contact with antibiotic regulatory authorities, and when information was shared, it was not properly targeted to the relevant audience. Lack of correct information sharing can hinder efforts to track and contain falsified and low-quality veterinary antibiotics in the market (18), as well as updates to policies, regulations, and antibiotic recalls (42). A study by Nayiga et al. (31) in Malawi, Tanzania and Uganda, reported concerns over little information sharing from regulatory authorities, with highly technical information often shared with non-technical audiences. Health and veterinary practitioners preferred general instructions to encourage prudent prescribing practices. There is need for regulatory authority to (1) develop online portal with real-time communication features like chat or help desk, (2) regularly update stakeholders on policy changes, new regulations, and enforcement activities. This can be done via newsletters, webinars, or stakeholder meetings, (3) provide constructive feedback and corrective actions for stakeholders who are not compliant, helping them align with regulations rather than imposing punitive measures, and (4) establish collaborative platforms or networks where stakeholders can share best practices and information, such as updated regulations or new training opportunities, can facilitate knowledge exchange and coordination.

Our study had limitations. Firstly, potential bias may have existed in the selection of study participants, as it was done with assistance from government veterinary officers and likely based on existing relationships. Secondly, only one local antibiotic manufacturer and three antibiotic wholesalers participated in the KIIs, potentially limiting variation in the information captured.

## 5 Conclusion

This study found that the distribution of veterinary antibiotics in Malawi involves both formal and informal pathways for importing and distributing veterinary medicines. Additionally, poor antibiotic prescribing and mishandling practices were reported across the distribution chain including a lack of awareness and appropriate antimicrobial stewardship training. There was also a lack of coordination and overlapping mandates among government authorities. There is need to strengthen regulations and enforcement mechanisms through having an antibiotic tracking systems, education campaigns, penalties for illegal distribution, and law enforcement involvement. Improved governance and coordination could be achieved through having clear regulatory mandates responsibilities, an inter-agency task force, and partnerships with international regulatory organizations. Correct storage and disposal of antibiotics could be addressed through a reverse logistics program, and training antibiotic shop attendants. Shops following best practices should be recognized, while better communication between regulatory authorities and stakeholders via online portals and real-time updates would promote prudent antibiotic use, facilitate knowledge exchange and better coordination.

## Data Availability

The original contributions presented in the study are included in the article/Supplementary material, further inquiries can be directed to the corresponding authors.
