# Supplementary material for: Understanding the veterinary antibiotic flow in Malawi: complexities, gaps and needs
Source: Front Vet Sci. 2024 Nov 20;11:1474307. doi: 10.3389/fvets.2024.1474307 (PMC11614805; doi:10.3389/fvets.2024.1474307)
Supplement: Supplementary file 1 [file Table_1.docx]

Supplementary Material

**Supplementary Table S1**

**Farmer Questionnaire**

**Section A:** **Background information**

| 1. Questionnaire ID |  |
| --- | --- |
| 1. District |  |
| 1. Location |  |
| 1. Date of Survey | DD/MM/YYYY |
| 1. Enumerator’s name |  |
| 1. Name of the respondent |  |
| 1. Sex of the respondent | 🞏1 Male, 🞏2 Female |
| 1. Age of respondent (years) | □0-30  □30-60  □Above 60 |
| 1. Number of years in practice | 1🞏 0-1 2🞏 1-4  3🞏4-10 4🞏 More than 10 |
| 1. Highest Qualification of the respondent | 🞏1 degree  🞏2 Diploma  🞏3 Certificate  🞏4 High School  🞏5 Primary school |
| 1. Which livestock species do you keep? | 🞏1 Pigs  🞏2 Cattle  🞏3 Sheep/goats  🞏4 Poultry  🞏5 Fish and Aquaculture  🞏5 Camels |
| 1. How many animals/birds to you keep? |  |
| 1. What do you do in case of diseases in your animals? | 🞏1 Self-treat  🞏2 Call a veterinarian/AHA  🞏3 Use Herbs  🞏4 Other (specify |

**Section B: Veterinary antibiotic supply chain**

| 1. Which antibiotics do you commonly use?   (Upon listing the antibiotics, researcher will classify the antibiotics to respective class of antibiotics) | 🞏1 Aminoglycosides  🞏2 β-lactam  🞏3 Fluoroquinolones  □4 Sulphonamides  🞏5 Tetracyclines  □6 Macrolides  🞏7 Polypeptides  □8 Others (specify) |
| --- | --- |
| 1. Where do you buy your antibiotics from | 🞏1.Veterinarian/ animal health practitioner  🞏2 Agrovet  🞏3 Distributing company  🞏4 Others (specify) |
| 1. What determines your choice of antibiotic you use | □ quantity/availability  □ quality/ brand  □ cost/price  □ condition  □ other (specify) |
| 1. Are you given any advice on their use? | □Yes  □No |
| 1. If yes, what kind of advice? | □1.Dosage  □2.Duration for use  □3.Route of administration  □4.Withdrawal period  □5.Other (specify) |
| 1. What is the most frequent way of buying the antibiotics? | □1 Proportion/measured.  🞏2 Whole package for whole course of treatment  🞏3 Other (specify) |
| 1. If you buy an antibiotic and does not work, what do you do | □ call a vet/AHA  □ purchase another antibiotic  □ use the same antibiotic at a higher dose  □ other (specify) |
| 1. How do you store your antibiotics | □ open shelves  □ refrigeration  □ cool boxes  □ cabinet  Other specify |
| 1. What do you do with expired antibiotics? | □1 use at a higher dose  🞏2 Discard off  🞏3 Return to the retailer  🞏5 Other (specify) |
| 1. Do you encounter any challenges when sourcing antibiotics | □Yes  □No |
| 1. If yes, which ones | □1.Availability  □2.Quality  □3.Quantity (doses)  □4. Cost  □5.Other (specify) |
| 1. Do you encounter counterfeit antibiotics | □ Yes  □ No |
| 1. If yes, what do you do |  |
| 1. Are you aware of the institutions that govern antibiotic use | □Yes  □No |
| 1. If yes, list them |  |
| 1. Are you aware of any policies governing antibiotic use/storage/handling | □ Yes  □ No |
| 1. If yes, which ones |  |
| 1. Where did you get this information from | □ internet/press  □ workshops/trainings  □background training  □other (specify) |
| 1. Do you think that the existing policies are effective | □ Yes  □ No |
| 1. If no, what should be done | □ frequent stakeholder trainings/workshop for awareness  □ stakeholder engagement in developing the policies  □ review of existing policies  □ other (specify |
| 1. Dis attend any stakeholder meetings the last 6 months?? | □ Yes  □ No |
| 1. If yes, who were the organizers |  |
| 1. Which other stakeholders attend such meetings |  |
| 1. If you don’t attend, why |  |

**Agrovet Questionnaire**

**Section A:** **Background information**

| Questionnaire ID |  |
| --- | --- |
| 1. District |  |
| 1. Location |  |
| 1. Date of Survey | DD/MM/YYYY |
| 1. Enumerator’s name |  |
| 1. Name of the respondent |  |
| 1. Sex of the respondent | 🞏1 Male, 🞏2 Female |
| 1. Age of respondent (years) |  |
| 1. Number of year working in the business / in practice | 1🞏0-1 2🞏1-4  3🞏4-10 4🞏More than 10 |
| 1. Qualification of the respondent | 🞏1 Bachelor of Veterinary Medicine (BVM)  🞏2 Bachelor of Science (BSc) in animal health  🞏3 Diploma in animal health  🞏4 Certificate in animal health  🞏5 others (specify) |
| 1. If other, have you ever received training or are you still training to dispense antibiotics for animal use? | □ Yes  □ No |
| 1. If yes, what is the duration of the training | □ 0-6months  □ 6-12months  □ over 12months |

**Section B: Veterinary antibiotic supply chain**

| 1. Which antibiotics do you commonly sell? (Upon listing the antibiotics, researcher will classify the antibiotics to their respective classes) | 🞏1 Aminoglycosides  🞏2 β-lactam  🞏3 Fluoroquinolones  □4 Sulphonamides  🞏5 Tetracyclines  □6 Macrolides  🞏7 Polypeptides  □8 Others (specify) |
| --- | --- |
| 1. Where do you source veterinary antibiotics from? | 🞏1.Manufacturer  🞏2 Distributors  🞏3 Retailer/ other agrovet  🞏4 Others (specify) |
| 1. What determines the choice of antibiotic that you stock | □1 demand  □2 cost/price  □3 storage method  □4 quantity/availability  □5 others (specify) |
| 1. What determines the price/cost of the antibiotics you sale | □1 quality/brand  □2 demand  □3 quantity/availability  □4 others (specify) |
| 1. What factors do you consider when recommending antibiotics to a customer? | □ history and clinical signs  □ based on prescription  □ based on laboratory report  □ other (specify) |
| 1. How do you sell antibiotics | 🞏1 On prescription only  🞏2 Without prescription  🞏3 Both  □4 Other (specify) |
| 1. For the case of antibiotics (and if without prescription), who decides on which antibiotic to give to the client | 🞏1 The client  🞏2 Myself (the drug stockist)  □3 a veterinarian/AHA  □4 Other (specify) |
| 1. If without prescription, what is the basis for deciding what antibiotic to give to your client | 🞏1 Symptoms as explained by the client  🞏2 Advice from a vet/AHA  🞏3 Laboratory test results provide by the client  □4 Other (specify) |
| 1. Do you give any advice to your customers on antibiotic use | □ Yes  □ No |
| 1. If yes, what kind of advice | □ dosage and duration  □ formulation  □ storage  □ withdrawal period  □ other (specify) |
| 1. How often do you give advice to a customer before they purchase antibiotics? | □ all the time  □ upon request  □ rarely  □ other (specify) |
| 1. What is the most frequent way of dispensing antibiotic? | 🞏1 Proportion/measurement depending on the client’s capacity to purchase.  🞏2 Whole package for whole course of treatment  🞏3 Other (specify) |
| 1. How do you store your antibiotics? | □ open shelves  □ under refrigeration  □ in cool boxes  □other (specify) |
| 1. Do you encounter any challenges when sourcing antibiotics | □Yes  □No |
| 1. If yes, which ones | □Availability  □Quality  □Quantity (doses)  □High cost  □Other (specify) |
| 1. Who are you main customers | □ poultry farmers  □ beef  □dairy farmers  □ pig farmers  □ other (specify) |
| 1. Do you Prescribe antibiotics to healthy animals as a form of prophylaxis | □ Yes  □ No |
| 1. If yes, to which clients mostly | □ poultry farmers  □ beef  □dairy farmers  □ pig farmers  □ other (specify) |
| 1. If you recommend or prescribe an antibiotic and the farmer returns to you and complains that it hasn't treated the disease, what do you do? | □ tell the client to increase dosage  □ recommend change of antibiotic  □ refer to a vet/AHA  □ Other (specify) |
| 1. How do dispose expired drugs | □1 sale at cheaper price/discount  □2 return to distributor  □3 discard  □4 others (specify) |
| 1. do you encounter counterfeit antibiotics | □ Yes  □ No |
| 1. If yes, what do you do |  |
| 1. Do you keep a record of all the antibiotics you dispense/prescribe? | □ Yes  □ No |
| 1. In case you don’t, why |  |
| 1. How often do you or the owner renew license to sell antibiotics for animal use? | □ annually  □ when expecting inspection  □ never |
| 1. Are you aware of the institutions that govern antibiotic use | □Yes  □No |
| 1. If yes, which ones |  |
| 1. How frequent do these institutions conduct monitoring and evaluation | □ very often  □ during routine inspections only  □ never  □ other (specify) |
| 1. Are you aware of any policies governing antibiotic use/storage/handling | □ Yes  □ No |
| 1. If yes, which one |  |
| 1. where did you get this information from | □ Education training  □ workshops  □ internet/ press  □other (specify) |
| 1. Are there existing channels for information sharing between you and the institutions | □ Yes  □ No |
| 1. If yes, which one |  |
| 1. Do you think that the existing policies are effective | □ Yes  □ No |
| 1. If no, what do you think can be done to make them more effective | □ frequent stakeholder trainings/workshop for awareness  □ stakeholder engagement in developing the policies.  □ review of existing policies  □ other (specify |
| 1. Do you attend any stakeholder meetings | □ Yes  □No |
| 1. If yes who organizes them |  |
| 1. Which other stakeholders attend such meetings |  |
| 1. In case you don’t attend, what is the reason |  |
| 1. Do you think there is sufficient interaction between you and the regulating bodies | □ Yes  □ No |
| 1. If no, what can be done |  |
| 1. How often do you renew your license? |  |

**Animal Health Practitioner Questionnaire**

**Section A:** **Background information**

| Questionnaire ID |  |
| --- | --- |
| 1. 1.District |  |
| 1. Location |  |
| 1. Date of Survey | DD/MM/YYYY |
| 1. Enumerator’s name |  |
| 1. Name of the respondent |  |
| 1. Sex of the respondent | 🞏1 Male, 🞏2 Female |
| 1. Age of respondent (years) | □0-30  □31-60  □Above 60 |
| 1. Number of year working in the business / in practice | 1🞏- 0-1 2🞏- 2-4  3🞏- 5-10 4🞏- More than 10 |
| 1. Qualification of the respondent | 🞏1 Bachelor of Veterinary Medicine (BVM)  🞏2 Bachelor of Science (BSc) in animal health  🞏3 Diploma in animal health  🞏4 Certificate in animal health |

**Section B: Veterinary antibiotic supply chain**

| 1. Which antibiotics do you commonly use | 🞏1 Aminoglycosides  🞏2 β-lactam  🞏3 Fluoroquinolones  □4 Sulphonamides  🞏5 Tetracyclines  □6 Macrolides  🞏7 Polypeptides  □8 Others (specify) |
| --- | --- |
| 1. Where do you source your antibiotics from | 🞏1.Manfacturer  🞏2 distributors  🞏3 Retailer/ agrovet  🞏4 Others (specify) |
| 1. What determines the choice of antibiotics that you use | □1 demand  □2 quantity/availability  □3 quality/brand  □4 storage method  □5 others (specify) |
| 1. Do you sell antibiotics | □Yes  □ No |
| 1. what is the basis for deciding what antibiotic to give to your client | 🞏1 Symptoms as explained by the client  🞏2 Own examinations  🞏3 Laboratory test results  □4 Other (specify) |
| 1. What is the most frequent way of dispensing antibiotic? | 🞏1 Proportion/measurement depending on the client’s capacity to purchase.  🞏2 Whole package for whole course of treatment  🞏3 Other (specify) |
| 1. How do you store your antibiotics | □Open shelves  □Refrigeration  □Cool boxes  □Other (specify) |
| 1. Do you give any advice to your clients | □ yes  □ No |
| 1. If yes, what kind of advice | □ dosage and duration  □ formulation  □ storage  □ withdrawal period  □ other (specify) |
| 1. How often do you do that | □ all the time  □ upon request  □ rarely  □ other (specify) |
| 1. Do you do follow up checks after treatment | □ Yes  □ No |
| 1. If yes how often | □ all the time  □ sometimes  □ never  □ other specify |
| 1. In case of treatment failure what do you do | □ increase dosage  □ change antibiotic  □ take sample for sensitivity  □ other (specify) |
| 1. What challenges do you experience when access antibiotic | 🞏1. Availability  □2 Qualities  □3 Quantities  □4 Costs  □5 Others (specify) |
| 1. Do you Prescribe antibiotics to healthy animals as a form of prophylaxis | □ yes  □ No |
| 1. If yes, to which clients | □ poultry farmers  □ beef  □dairy farmers  □ pig farmers  □ other (specify) |
| 1. Do you keep a record of all the antibiotics you dispense/prescribe? | □ Yes  □ No |
| 1. If yes, what’s the quantity of antibiotics you sell/prescribe per month | □0-100 kgs  □100-200 kgs  □200-300 kgs  □ above 300 kgs |
| 1. In case you don’t, why |  |
| 1. How do you dispose expired antibiotics | □1 sale at discounted prices  □2 Use at higher dose  □3 Discard  □4 others (specify) |
| 1. Do you encounter counterfeit antibiotics | □ Yes  □ No |
| 1. If yes, what do you do |  |
| 1. What is your role along the supply chain | □ 1 Treatment only  □ 2 Consultation and treatment  □ 3 Selling of antibiotic, consultation, and treatment |
| 1. Are you aware of the institutions that govern antibiotic use | □ Yes  □ No |
| 1. If yes, list them |  |
| 1. Are you aware of any policies governing antibiotic use/storage/handling | □ yes  □ No |
| 1. If yes, which one |  |
| 1. Where did you get this information from | □ background training  □ workshops  □ internet/ press  □other (specify) |
| 1. Are there existing channels for information sharing between you and the institutions | □ Yes  □ No |
| 1. If yes, which ones |  |
| 1. Do you think that the existing policies are effective | □ Yes  □ No |
| 1. If no, what do you think can be done to make them more effective | □ frequent stakeholder trainings/workshop for awareness  □ stakeholder engagement in developing the policies  □ review of existing policies  □ other (specify) |
| 1. Do you attend any stakeholder meetings | □ Yes  □ No |
| 1. If yes, who organizes them |  |
| 1. Which other stakeholders attend such meetings |  |
| 1. In case you don’t attend, what is the reason |  |
| 1. Do you think there is sufficient interaction between you and the regulating bodies | □ Yes  □ No |
| 1. If no, what can be done |  |

**Focus Group discussions with Farmers**

**Section A: Participant demographics**

| 1. Name of the participant |  |
| --- | --- |
| 1. Sex of the participant | 🞏1 Male  🞏2 Female |
| 1. Age of participant (years) | □0-30  □31-60  □Above 60 |
| 1. Number of year working in the business / in practice | 1🞏- 0-1 2🞏- 2-4  3🞏- 5-10 4🞏- More than 10 |
| 1. Qualification of the participant | 🞏1 Degree  🞏2 Diploma  🞏3 Certificate  🞏4 Secondary  □5 Primary |
| 1. Previous training on antimicrobial resistance or use |  |

**Section B: Animal health/Antibiotic use**

1. What do you do when you encounter diseases on your farm?
2. How often do you use/buy antibiotics?
3. Which antibiotics do you commonly use?
4. What are the reasons for using antibiotics and what informs the choice?
5. Where do buy the antibiotics from/reasons for this source?
6. Is this source reliable?
7. How do you store your antibiotics?
8. What do you do when antibiotics fail to work?
9. Do you complete the recommended dosage for antibiotics?
10. What challenges do you face in accessing antibiotics?

**Section C: Access to animal health services**

1. Have you ever sent samples of suspected disease to the laboratory for diagnosis in the last 12 months?
2. If yes, where did you send your samples? government or private lab??
3. Were you satisfied with the service?
4. Where do you get advice regarding the use of antibiotics?
5. What kind of advice are you given regarding antibiotic use?
6. How often do you call your vet/animal health practitioner?
7. How often do you encounter counterfeit antibiotics?
8. What do you do with them?
9. What do you think contributes to the presence of counterfeit antibiotics?
10. How often do you attend trainings?
11. Who organizes such training?

**Section D: Governances and interactions**

1. What is your take on the effectiveness of the existing policies on antibiotic use?
2. Which other stakeholders do you interact with, and what type of interaction?

**Focus Group discussions with agrovet owners/vendor/workers**

**Section A: Participants demographics**

| 1. Name of the participant |  |
| --- | --- |
| 1. Sex of the participant | 🞏1 Male  🞏2 Female |
| 1. Age of participant (years) | □0-30  □31-60  □Above 60 |
| 1. Number of year working in the business / in practice | 1🞏- 0-1 2🞏- 2-4  3🞏- 5-10 4🞏- More than 10 |
| 1. Qualification of the participant | 🞏1 Bachelor of Veterinary Medicine (BVM)  🞏2 Bachelor of Science (BSc) in animal health  🞏3 Diploma in animal health  🞏4 Certificate in animal health  □5 others(specify) |

**Section B: Animal health/Antibiotic sale**

1. Which antibiotics do you commonly sale?
2. Where do source antibiotics from?
3. What informs the choice of antibiotics to sale?
4. What dictates the price of antibiotics?
5. How do you store your antibiotics?
6. Which are the common practices regarding antibiotic use?
7. Which challenges do you encounter in accessing antibiotics?
8. Who are your main customers?
9. How do you dispose off expired antibiotics?

**Section C: Access to animal health services**

1. What kind of advice do you give to clients/farmers regarding antibiotic use?
2. Do you offer vet lab diagnostics or use?
3. How often do you encounter counterfeit antibiotics?
4. Do you know the reporting mechanism for counterfeits?
5. What do you think contributes to the presence of counterfeit antibiotics?
6. What are the benefits of record keeping on antibiotic sale/use?
7. Which other stakeholders do you interact with and what type of interaction?

**Section D: Governance structure and interactions**

1. Who oversees regulation of antibiotic use?
2. How often do these bodies conduct monitoring and evaluation and what is the impact?
3. What are some of the benefits of having policies on antibiotic use?
4. What is your take on the effectiveness of the existing policies on antibiotic use?
5. What challenges affect the functionality of the existing policies?
6. What do you think should be done to improve the interaction between stakeholders and the relevant regulating bodies?
7. What is the cost of license renewal?
8. How often do you renew the license??

**Focus Group discussions with animal health practitioners**

**Section A: Participants demographics**

| 1. Name of the participant |  |
| --- | --- |
| 1. Sex of the participant | 🞏1 Male  🞏2 Female |
| 1. Age of participant (years) | □0-30  □31-60  □Above 60 |
| 1. Number of year working in the business / in practice | 1🞏- 0-1 2🞏- 2-4  3🞏- 5-10 4🞏- More than 10 |
| 1. Qualification of the participant | 🞏1 Bachelor of Veterinary Medicine (BVM)  🞏2 Bachelor of Science (BSc) in animal health  🞏3 Diploma in animal health  🞏4 Certificate in animal health  Others (specify) |

**Section B: Animal health/Antibiotic use**

1. Which antibiotics do you commonly sale/use?
2. Where do you source the antibiotics that you sale/use from?
3. What informs the choice of antibiotics that you sale/use?
4. What dictates the price of antibiotics that you sale/use?
5. Which challenges do you encounter in accessing antibiotics?
6. Who are your main customers?
7. Which are the common practices regarding antibiotic use?

**Section C: Animal health services**

1. What kind of advice do you give to clients/farmers regarding antibiotic use?
2. What are the benefits of proper record keeping on antibiotic sale/use?
3. How often do you encounter counterfeit antibiotics?
4. What do you think contributes to the presence of counterfeit antibiotics?
5. How often do you attend training?
6. How have these trainings benefited you?
7. Who organizes such training?
8. Which stakeholders do you interact with and what type of interaction?
9. What are your roles along the antibiotic supply chain?

**Section D: Governance structure and interactions**

1. Who oversees regulation of antibiotic use?
2. How often do these bodies conduct monitoring and evaluation and what is the impact?
3. What are some of the benefits of having policies on antibiotic use?
4. What is your take on the effectiveness of the existing policies on antibiotic use?
5. What challenges affect the functionality of the existing policies?
6. What is your take on the private public partnership pertaining to antibiotic policy implementation?
7. What do you think should be done to improve the interaction between stakeholders and the relevant regulating bodies?

**Key Informant Interview Guide**

1. Discuss finds from FGDs and seek more clarification on areas that need to be
2. What’s your role along the antibiotic supply chain?
3. How often do you conduct monitoring and evaluation and what is the impact?
4. What are some of the policies on antibiotic use?
5. What is your take on the effectiveness of the existing policies on antibiotic use and the level of implementation?
6. What is your take on the private public partnership pertaining to antibiotic policy implementation?
7. What is the level of interaction between you and the stakeholders on matters of policy development and implementation?
8. What do you think should be done to improve the interaction between relevant regulatory bodies and stakeholders?

**Results**

**Table 1:** The social-demographic characteristics of pharmaceutical businesses attendants interviewed in the study.

| **Social Demographic Characteristics of Pharmaceutical businesses** | | | |
| --- | --- | --- | --- |
| **Variable** | **Responses** | **Total (35)** | **%** |
| 1. District | 1. Mzuzu | 3 | 8.6 |
|  | 1. Lilongwe | 20 | 57.1 |
|  | 1. Blantyre | 12 | 34.3 |
| 1. Sex of the respondent | 1. Male | 21 | 60.0 |
|  | 1. Female | 14 | 40.0 |
| 1. Age of respondent (years) | 1. 0-17 | 0 | 0.0 |
|  | 1. 18-30 | 25 | 71.4 |
|  | 1. 31-40 | 6 | 17.1 |
|  | 1. 41-50 | 2 | 5.7 |
|  | 1. Above 50 | 2 | 5.7 |
| 1. Number of years in business | 1. 0 to 1 | 8 | 22.9 |
|  | 1. 1 to 4 | 18 | 51.4 |
|  | 1. 4 to 10 | 4 | 11.4 |
|  | 1. More than 10 | 4 | 11.4 |
| 1. Qualification of the respondent | 1. Bachelor of Veterinary Medicine (BVM) | 1 | 2.9 |
|  | 1. Bachelor of Science (BSc) in animal science | 1 | 2.9 |
|  | 1. Diploma in animal health | 16 | 45.7 |
|  | 1. Certificate in animal health | 3 | 8.6 |
|  | 1. others (specify) | 14 | 40.0 |
| 1. If other, have you ever received training or are you still training to dispense antibiotics for animal use? | 1. Yes | 8 | 22.9 |
|  | 1. No | 6 | 17.1 |
| 1. If yes, what is the duration of the training | 1. 0-6months | 0 | 0.0 |
|  | 1. 6-12months | 0 | 0.0 |
|  | 1. over 12months | 0 | 0.0 |

**Table 2:** Access and challenges to Antibiotics Stocked by Wholesalers and Retailers

| **Access and challenges to Antibiotics Stocked by Wholesalers and Retailers** | | | |
| --- | --- | --- | --- |
| **Variable** | **Responses** | **Total (35)** | **%** |
| 1. Which antibiotics classes do you commonly sell? | 1. Aminoglycosides | 9 | 25.7 |
|  | 2. β-lactam | 21 | 60.0 |
|  | 3 Fluoroquinolones | 7 | 20.0 |
|  | 4 Sulphonamides | 15 | 42.9 |
|  | 5 Tetracyclines | 30 | 85.7 |
|  | 6 Macrolides | 2 | 5.7 |
|  | 7 Polypeptides | 3 | 8.6 |
|  | 8 Others (specify) | 0 | 0.0 |
| 1. Where do you source veterinary antibiotics from? | 1.Manufacturer | 4 | 11.4 |
|  | 2 Distributors | 13 | 37.1 |
|  | 3 Retailer | 7 | 20.0 |
|  | 4 Imports | 18 | 51.4 |
| 1. What determines the choice of antibiotic that you stock | 1 demand | 33 | 94.3 |
|  | 2 cost/price | 1 | 2.9 |
|  | 3 storage method | 0 | 0.0 |
|  | 4 quantity/availability | 1 | 2.9 |
|  | 5 others (specify) | 0 | 0.0 |
| 1. What determines the price/cost of the antibiotics sold | 1 quality/brand | 4 | 11.4 |
|  | 2 demands | 1 | 2.9 |
|  | 3 quantity/availability | 1 | 2.9 |
|  | 4 others (specify) | 29 | 82.9 |
| 1. What factors do you consider when recommending antibiotics to a customer? | 1. history and clinical signs | 31 | 88.6 |
|  | 1. based on prescription | 2 | 5.7 |
|  | 1. based on laboratory report | 0 | 0.0 |
|  | 1. other (specify) | 4 | 11.4 |
| 1. How do you sell antibiotics | 1 On prescription only | 4 | 11.4 |
|  | 2 Without prescription | 33 | 94.3 |
|  | 3 Both | 1 | 2.9 |
|  | 4 Other (specify) |  | 0.0 |
| 1. Who decides on which antibiotic to give to the client | 1 The client | 6 | 17.1 |
|  | 2 Myself (the drug stockist) | 20 | 57.1 |
|  | 3 a veterinarian/AHA | 8 | 22.9 |
|  | 4 Other (specify) | 1 | 2.9 |
| 1. If without prescription, what is the basis for deciding what antibiotic to give to your client | 1 Symptoms as explained by the client | 30 | 85.7 |
|  | 2 Advice from a vet/AHA | 4 | 11.4 |
|  | 3 Laboratory test results provide by the client | 1 | 2.9 |
|  | 4 Other (specify) | 0 | 0.0 |
| 1. Do you give any advice to your customers on antibiotic use | 1. Yes | 31 | 88.6 |
|  | 1. No | 4 | 11.4 |
| 1. If yes, what kind of advice | 1. dosage and duration | 23 | 65.7 |
|  | 1. formulation | 3 | 8.6 |
|  | 1. storage | 1 | 2.9 |
|  | 1. withdrawal period | 3 | 8.6 |
|  | 1. other (specify) | 1 | 2.9 |
| 1. How often do you give advice to a customer before they purchase antibiotics? | 1. All the time | 22 | 62.9 |
|  | 1. upon request | 8 | 22.9 |
|  | 1. rarely | 1 | 2.9 |
|  | 1. other (specify) | 0 | 0.0 |
| 1. What is the most frequent way of dispensing antibiotic? | 1. Proportion/measurement depending on the client’s capacity to purchase | 4 | 11.4 |
|  | 1. Whole package for whole course of treatment | 31 | 88.6 |
|  | 1. Other (specify) | 0 | 0.0 |
| 1. How do you store your antibiotics? | 1. open shelves | 31 | 88.6 |
|  | 1. under refrigeration | 0 | 0.0 |
|  | 1. in cool boxes | 1 | 2.9 |
|  | 1. other (specify) | 3 | 8.6 |
| 1. Do you encounter any challenges when sourcing antibiotics | 1. Yes | 24 | 68.6 |
|  | 1. No | 11 | 31.4 |
| 1. If yes, which ones | 1. Availability | 1 | 2.9 |
|  | 1. Quality | 1 | 2.9 |
|  | 1. Quantity (doses) | 4 | 11.4 |
|  | 1. High cost | 16 | 45.7 |
|  | 1. Other (specify) |  | 0.0 |
| 1. Who are you main customers | 1. poultry farmers | 27 | 77.1 |
|  | 1. beef | 12 | 34.3 |
|  | 1. dairy farmers | 7 | 20.0 |
|  | 1. pig farmers | 19 | 54.3 |
|  | 1. Aquaculture | 2 | 5.7 |
| 1. Do you Prescribe antibiotics to healthy animals as a form of prophylaxis | 1. Yes | 24 | 68.6 |
|  | 1. No | 11 | 31.4 |
| 1. If yes, to which clients mostly | 1. poultry farmers | 19 | 54.3 |
|  | 1. beef | 2 | 5.7 |
|  | 1. dairy farmers | 1 | 2.9 |
|  | 1. pig farmers | 6 | 17.1 |
|  | 1. Sheep and goats Farming | 5 | 14.3 |
| 1. If you recommend or prescribe an antibiotic and the farmer returns to you and complains that it hasn't treated the disease, what do you do? | 1. tell the client to increase dosage | 3 | 8.6 |
|  | 1. recommend change of antibiotic | 22 | 62.9 |
|  | 1. refer to a vet/AHA | 4 | 11.4 |
|  | 1. Other (specify) | 5 | 14.3 |
| 1. How do dispose expired drugs | 1. 1 sale at cheaper price/discount | 2 | 5.7 |
|  | 1. 2 returns to distributor | 1 | 2.9 |
|  | 1. 3 discards | 19 | 54.3 |
|  | 1. 4 others (specify) | 13 | 37.1 |
| 1. do you encounter counterfeit antibiotics | 1. Yes | 7 | 20.0 |
|  | 1. No | 28 | 80.0 |
| 1. Do you keep a record of all the antibiotics you dispense/prescribe? | 1. Yes | 32 | 91.4 |
|  | 1. No | 3 | 8.6 |
| 1. How often do you or the owner renew license to sell antibiotics for animal use? | 1. annually | 12 | 34.3 |
|  | 1. when expecting inspection | 21 | 60.0 |
|  | 1. never | 2 | 5.7 |
| 1. Are you aware of the institutions that govern antibiotic use | 1. Yes | 26 | 74.3 |
|  | 1. No | 9 | 25.7 |
| 1. If yes, which ones | 1. PMRA | 26 | 74.3 |
|  | 1. DAHLD | 2 | 5.7 |
|  | 1. Don’t know | 9 | 25.7 |
| 1. How frequent do these institutions conduct monitoring and evaluation | 1. very often | 6 | 17.1 |
|  | 1. during routine inspections only | 25 | 71.4 |
|  | 1. never | 4 | 11.4 |
|  | 1. other | 0 | 0.0 |
| 1. Are you aware of any policies governing antibiotics | 1. Yes | 20 | 57.1 |
|  | 1. No | 15 | 42.9 |
| 1. where did you get this information from | 1. Education training | 10 | 28.6 |
|  | 1. workshops | 0 | 0.0 |
|  | 1. internet/ press | 4 | 11.4 |
|  | 1. other | 6 | 17.1 |
| 1. Are there existing channels for information sharing between you and the institutions | 1. Yes | 14 | 40.0 |
|  | 1. No | 21 | 60.0 |
| 1. Do you think that the existing policies are effective | 1. Yes | 9 | 25.7 |
|  | 1. No | 11 | 31.4 |
| 1. If no, what do you think can be done to make them more effective | 1. frequent stakeholder trainings/ workshop for awareness | 11 | 31.4 |
|  | 1. stakeholder engagement in developing the policies. | 6 | 17.1 |
|  | 1. review of existing policies | 1 | 2.9 |
|  | 1. others | 2 | 5.7 |
| 1. Do you attend any stakeholder meetings? | 1. Yes | 10 | 28.6 |
|  | 1. No | 25 | 71.4 |
| 1. Do you think there is sufficient interaction between you and the regulating bodies? | 1. Yes | 10 | 28.6 |
|  | 1. No | 25 | 71.4 |

**Table 3:** Social Demographic Characteristics of Animal Health Practitioners

| **Social Demographic Characteristics of Animal Health Practitioners** | | | |
| --- | --- | --- | --- |
| **Variable** | **Responses** | **Total (38)** | **%** |
| 1. District | 1. Mzuzu | 2 | 5.3 |
|  | 1. Lilongwe | 27 | 71.1 |
|  | 1. Blantyre | 9 | 23.7 |
| 1. Sex of the respondent | 1. Male | 12 | 31.6 |
|  | 1. Female | 26 | 68.4 |
| 1. Age of respondent (years) | 1. 0-17 | 0 | 0.0 |
|  | 1. 18-30 | 5 | 13.2 |
|  | 1. 31-40 | 18 | 47.4 |
|  | 1. 41-50 | 9 | 23.7 |
|  | 1. Above 50 | 6 | 15.8 |
| 1. Number of years in practice | 1. 0 to 1 | 0 | 0.0 |
|  | 1. 1 to 4 | 6 | 15.8 |
|  | 1. 4 to 10 | 12 | 31.6 |
|  | 1. More than 10 | 20 | 52.6 |
| 1. Qualification of the respondent | 1. Bachelor of Veterinary Medicine | 10 | 26.3 |
|  | 1. Bachelor of animal science | 2 | 5.3 |
|  | 1. Diploma in animal health | 10 | 26.3 |
|  | 1. Certificate in animal health | 15 | 39.5 |

**Table 4: Knowledge, Practices, and Awareness About AMU and Perception of Related Policies on AHPs**

| **Knowledge, Practices, and Awareness About AMU and Perception of Related Policies on**  **AHPs** | | | |
| --- | --- | --- | --- |
| **Variable** | **Responses** | **Total (38)** | **%** |
| 1. Which antibiotic classes do you commonly buy/ use? | 1. Aminoglycosides | 9 | 23.7 |
|  | 1. β-lactam | 21 | 55.3 |
|  | 1. Fluoroquinolones | 9 | 23.7 |
|  | 1. Sulphonamides | 10 | 26.3 |
|  | 1. Tetracyclines | 34 | 89.5 |
|  | 1. Macrolides | 2 | 5.3 |
|  | 1. Polypeptides | 0 | 0.0 |
| 1. Where do you source your antibiotics from? | 1. Local Manufacturer | 1 | 2.6 |
|  | 1. Distributors | 3 | 7.9 |
|  | 1. Retailer/ other agrovet | 36 | 94.7 |
|  | 1. Imports | 3 | 7.9 |
|  | 1. Others | 1 | 2.6 |
| 1. What determines the choice of antibiotics that you use? | 1. demand | 35 | 92.1 |
|  | 1. quantity/availability | 1 | 2.6 |
|  | 1. quality/brand | 1 | 2.6 |
|  | 1. storage method | 0 | 0.0 |
|  | 1. others | 1 | 2.6 |
| 1. Do you sell antibiotics? | 1. Yes | 8 | 21.1 |
|  | 1. No | 30 | 78.9 |
| 1. what is the basis for deciding what antibiotic to give to your client? | 1. Symptoms as explained by the client | 28 | 73.7 |
|  | 1. Own examinations | 5 | 13.2 |
|  | 1. Laboratory test results | 5 | 13.2 |
| 1. What is the most frequent way of dispensing antibiotic? | 1. Proportion/measurement depending on the client’s capacity to purchase. | 12 | 31.6 |
|  | 1. Whole package for whole course of treatment | 8 | 21.1 |
|  | 1. Others | 10 | 26.3 |
| 1. How do you store your antibiotics | 1. Open shelves | 12 | 31.6 |
|  | 1. Refrigeration | 1 | 2.6 |
|  | 1. Cool boxes | 14 | 36.8 |
|  | 1. Others | 12 | 31.6 |
| 1. Do you give any advice to your clients | 1. yes | 37 | 97.4 |
|  | 1. No | 1 | 2.6 |
| 1. If yes, what kind of advice | 1. dosage and duration | 16 | 42.1 |
|  | 1. formulation | 1 | 2.6 |
|  | 1. storage | 2 | 5.3 |
|  | 1. withdrawal period | 6 | 15.8 |
|  | 1. other | 12 | 31.6 |
| 1. How often do you do that | 1. all the time | 32 | 84.2 |
|  | 1. upon request | 4 | 10.5 |
|  | 1. rarely | 1 | 2.6 |
| 1. Do you do follow up checks after treatment | 1. Yes | 36 | 94.7 |
|  | 1. No | 2 | 5.3 |
| 1. If yes how often | 1. all the time | 22 | 57.9 |
|  | 1. sometimes | 14 | 36.8 |
|  | 1. never | 0 | 0.0 |
| 1. In case of treatment failure what do you do | 1. increase dosage | 4 | 10.5 |
|  | 1. change antibiotic | 18 | 47.4 |
|  | 1. take sample for sensitivity | 10 | 26.3 |
|  | 1. other | 6 | 15.8 |
| 1. What challenges do you experience when access antibiotic | 1. Availability | 23 | 60.5 |
|  | 1. Qualities | 5 | 13.2 |
|  | 1. Quantities | 0 | 0.0 |
|  | 1. Costs | 10 | 26.3 |
| 1. Do you Prescribe antibiotics to healthy animals as a form of prophylaxis | 1. yes | 18 | 47.4 |
|  | 1. No | 20 | 52.6 |
| 1. If yes, to which clients | 1. poultry farmers | 12 | 31.6 |
|  | 1. beef | 6 | 15.8 |
|  | 1. dairy farmers | 5 | 13.2 |
|  | 1. pig farmers | 7 | 18.4 |
| 1. Do you keep a record of all the antibiotics you dispense/prescribe? | 1. Yes | 29 | 76.3 |
|  | 1. No | 9 | 23.7 |
| 1. If yes, what’s the quantity of antibiotics you sell/prescribe per month | 1. 0-100 kgs | 36 | 94.7 |
|  | 1. 100-200 kgs | 1 | 2.6 |
|  | 1. 200-300 kgs | 0 | 0.0 |
|  | 1. above 300 kgs | 1 | 2.6 |
| 1. 20.   How do you dispose expired antibiotics | 1. sale at discounted prices | 1 | 2.6 |
|  | 1. Use at higher dose | 0 | 0.0 |
|  | 1. Discard | 33 | 86.8 |
|  | 1. others | 4 | 10.5 |
| 1. Do you encounter counterfeit antibiotics | 1. Yes | 12 | 31.6 |
|  | 1. No | 26 | 68.4 |
| 1. What is your role along the supply chain | 1. Treatment only | 6 | 15.8 |
|  | 1. Consultation and treatment | 25 | 65.8 |
|  | 1. Selling of antibiotic, consultation, and treatment | 7 | 18.4 |
| 1. Are you aware of the institutions that govern antibiotic use | 1. Yes | 25 | 65.8 |
|  | 1. No | 13 | 34.2 |
| 1. If yes, list them | 1. PMRA | 25 | 65.8 |
|  | 1. DAHLD | 2 | 5.3 |
|  | 1. Don’t know | 12 | 31.6 |
| 1. Are you aware of any policies governing antibiotic use/storage/handling | 1. yes | 18 | 47.4 |
|  | 1. No | 20 | 52.6 |
| 1. Where did you get this information from | 1. background training | 10 | 26.3 |
|  | 1. workshops | 3 | 7.9 |
|  | 1. internet/ press | 4 | 10.5 |
|  | 1. others | 1 | 2.6 |
| 1. Are there existing channels for information sharing between you and the institutions | 1. Yes | 8 | 21.1 |
|  | 1. No | 17 | 44.7 |
| 1. Do you think that the existing policies are effective | 1. Yes | 3 | 7.9 |
|  | 1. No | 15 | 39.5 |
| 1. If no, what do you think can be done to make them more effective | 1. frequent stakeholder trainings/workshop for awareness |  | 0.0 |
|  | 1. stakeholder engagement in developing the policies |  | 0.0 |
|  | 1. review of existing policies |  | 0.0 |
| 1. Do you attend any stakeholder meetings | 1. Yes | 13 | 34.2 |
|  | 1. No | 25 | 65.8 |
| 1. Do you think there is sufficient interaction between you and the regulating bodies | 1. Yes | 3 | 7.9 |
|  | 1. No | 35 | 92.1 |

**Table 5:** Social Demographic Characteristics of Livestock Farmers

| **Variable** | **Responses** | **N=107** | **%** |
| --- | --- | --- | --- |
| **Social Demographic Characteristics of Livestock Farmers** | | | |
| 1. District | 1. Mzuzu | 22 | 20.6 |
|  | 1. Lilongwe | 45 | 42.1 |
|  | 1. Blantyre | 40 | 37.4 |
| 1. Sex of the respondent | 1. Male | 62 | 57.9 |
|  | 1. Female | 45 | 42.1 |
| 1. Age of respondent (years) | 1. 0-30 | 30 | 28.0 |
|  | 1. 30-60 | 68 | 63.6 |
|  | 1. Above 60 | 9 | 8.4 |
| 1. Number of years in practice | 1. 0 to 1 | 8 | 7.5 |
|  | 1. 1 to 4 | 34 | 31.8 |
|  | 1. 4 to 10 | 24 | 22.4 |
|  | 1. More than 10 | 41 | 38.3 |
| 1. Highest Qualification of the respondent | 1. Primary Education | 75 | 70.1 |
|  | 1. Secondary Education | 31 | 29.0 |
|  | 1. Tertiary Education | 1 | 0.9 |
| 1. Which livestock species do you keep? | 1. Pigs | 23 | 21.5 |
|  | 1. Cattle | 40 | 37.4 |
|  | 1. Sheep/goats | 21 | 19.6 |
|  | 1. Poultry | 22 | 20.6 |
|  | 1. Fish and Aquaculture | 3 | 2.8 |
| 1. What do you do in case of diseases in your animals? | 1. Self-treat | 9 | 8.4 |
|  | 1. Call a veterinarian | 79 | 73.8 |
|  | 1. Use Herbs | 7 | 6.5 |
|  | 1. Other | 12 | 11.2 |

**Table 6:** Knowledge, Practices, and Awareness About AMU and Perception of Related Policies among Livestock Farmers

| **Knowledge, Practices, and Awareness About AMU and Perception of Related Policies among Livestock Farmers** | | | |
| --- | --- | --- | --- |
| **Variable** | **Responses** | **N=107** | **%** |
| 1. Which antibiotic classes do you commonly use? | 1. Aminoglycosides | 3 | 2.8 |
|  | 1. β-lactam | 9 | 8.4 |
|  | 1. Fluoroquinolones | 0 | 0.0 |
|  | 1. Sulphonamides | 0 | 0.0 |
|  | 1. Tetracyclines | 13 | 12.1 |
|  | 1. Macrolides | 0 | 0.0 |
|  | 1. Polypeptides | 0 | 0.0 |
|  | 1. Don’t know | 82 | 76.6 |
| 1. Where do you buy your antibiotics from? | 1. Animal health practitioner | 6 | 5.6 |
|  | 1. Agrovet | 11 | 10.3 |
|  | 1. Distributing company | 0 | 0.0 |
|  | 1. Never bought | 90 | 84.1 |
| 1. What determines your choice of antibiotic you use? | 1. quantity/availability | 0 | 0.0 |
|  | 1. quality/ brand | 3 | 2.8 |
|  | 1. cost/price | 0 | 0.0 |
|  | 1. condition | 7 | 6.5 |
|  | 1. Don’t know | 97 | 90.7 |
| 1. Are you given any advice on their use? | 1. Yes | 29 | 27.1 |
|  | 1. No | 78 | 72.9 |
| 1. What is the most frequent way of buying the antibiotics? | 1. Proportion/measured | 3 | 2.8 |
|  | 1. Whole package | 20 | 18.7 |
|  | 1. Other | 84 | 78.5 |
| 1. If you buy an antibiotic and does not work, what do you do? | 1. call a vet | 33 | 30.8 |
|  | 1. purchase another antibiotic | 3 | 2.8 |
|  | 1. use a higher dose | 0 | 0.0 |
|  | 1. other | 70 | 65.4 |
| 1. How do you store your antibiotics? | 1. open shelves | 4 | 3.7 |
|  | 1. refrigeration | 0 | 0.0 |
|  | 1. cool boxes | 3 | 2.8 |
|  | 1. cabinet | 4 | 3.7 |
|  | 1. Other | 96 | 89.7 |
| 1. What do you do with expired antibiotics? | 1. use at a higher dose | 0 | 0.0 |
|  | 1. Bins | 14 | 13.1 |
|  | 1. Return to the retailer | 0 | 0.0 |
|  | 1. Pit latrines | 93 | 86.9 |
| 1. Do you encounter any challenges when sourcing antibiotics? | 1. Yes | 41 | 38.3 |
|  | 1. No | 66 | 61.7 |
| 1. If yes, which ones? | 1. Availability | 10 | 9.3 |
|  | 1. Quality | 0 | 0.0 |
|  | 1. Quantity (doses) | 0 | 0.0 |
|  | 1. Cost | 23 | 21.5 |
|  | 1. No challenges | 66 | 61.7 |
| 1. Do you encounter counterfeit antibiotics? | 1. Yes | 3 | 2.8 |
|  | 1. No | 103 | 96.3 |
| 1. Are you aware of the institutions that govern antibiotic use? | 1. Yes | 3 | 2.8 |
|  | 1. No | 103 | 96.3 |
| 1. Are you aware of any policies governing antibiotic use/storage/handling? | 1. Yes | 14 | 13.1 |
|  | 1. No | 93 | 86.9 |
| 1. Where did you get this information from? | 1. internet/press | 1 | 0.9 |
|  | 1. workshops/trainings | 4 | 3.7 |
|  | 1. background training | 4 | 3.7 |
|  | 1. other | 93 | 86.9 |
| 1. Do you think that the existing policies are effective? | 1. Yes | 5 | 4.7 |
|  | 1. No | 102 | 95.3 |
| 1. If no, what should be done? | 1. frequent stakeholder trainings/ workshop for awareness | 77 | 72.0 |
|  | 1. stakeholder engagement in developing the policies | 4 | 3.7 |
|  | 1. review of existing policies | 1 | 0.9 |
|  | 1. other | 25 | 23.4 |
| 1. Did you attend any stakeholder meetings the last 6 months? | 1. Yes | 16 | 15.0 |
|  | 1. No | 91 | 85.0 |
